# Mind the Gap: Predictors of Osteoporosis Treatment Following Fragility Fracture in Parkinsonism

**DOI:** 10.1002/mdc3.70652

**Published:** 2026-05-10

**Authors:** Katie C. Naylor, Emily J. Henderson, Emma Tenison

**Affiliations:** ^1^ Ageing and Movement Research Group, Population Health Sciences, Bristol Medical School University of Bristol Bristol UK; ^2^ Older Peoples Unit Royal United Hospital NHS Foundation Trust Bath UK

**Keywords:** BMD, fracture, osteoporosis, parkinsonism, Parkinson's disease

## Abstract

**Background:**

Fracture risk is increased in Parkinson's yet this risk is often not addressed.

**Objectives:**

Our objective was to study the extent to which osteoporosis was treated, and predictors of treatment in a large representative cohort with parkinsonism.

**Methods:**

The clinical practice research datalink (CPRD) GOLD data contains anonymized UK primary care data. We identified fragility fractures (hip, vertebrae, wrist/distal radius, humerus, rib, pelvis and unspecified osteoporotic) amongst prevalent parkinsonism patients between 2010 and 2019, and ascertained whether bone protective medications and/or vitamin D/calcium supplements were prescribed in the pre‐fracture period or in the 48‐weeks post‐fracture. Logistic regression was used to determine predictors of prescriptions.

**Results:**

There were 21,581 people with parkinsonism (mean age 74.7 ± 9.8 years, 40% female), with a mean of 3.1 ± 2.5 years of available data. One thousand eight hundred twenty‐three experienced at least one fragility fracture. Prior to a fragility fracture 12% had a prescription for bone protective medication. This increased to 38% and 53% in the 16 and 48‐weeks post‐fracture, respectively. 24% already had a prescription for vitamin D/calcium which increased to 73% at 48‐weeks post‐fracture. Female gender (OR = 2.06 [1.69–2.51], *p* < 0.001) and sustaining a vertebral fracture (OR = 2.06 [1.53–2.77], *p* < 0.001) increased the odds of bone protection prescriptions. However, sustaining a rib fracture (OR = 0.34 [0.19–0.60], *p* < 0.001) or residing in a care home (OR = 0.58 [0.38–0.88], *p* = 0.01) decreased the odds.

**Conclusions:**

There is an osteoporosis treatment gap in Parkinson's rendering an already high‐risk population at further risk of fracture. Men and those with rib fractures are two groups who are least likely to receive potentially beneficial treatment.

In Parkinson's disease (PD), lower bone mineral density (BMD) and increased incidence of falls lead to an increased fragility fracture risk.^1^, ^2^ Compared to age and gender‐matched controls, people with PD are at 2‐fold increased risk of osteoporotic fracture, and 3‐fold increased risk for hip fracture.^3^ Furthermore, people with PD are more likely to develop complications^4^, ^5^ or have difficulty regaining mobility^6^ following hip fracture and mortality is over twice that of age‐matched controls.^7^

In Parkinson's care bone health and osteoporosis prevention and treatment are often overlooked. In their 2020 UK‐wide national audit, Parkinson's UK reported that, of 1131 patients, 73% did not have an up‐to‐date fracture risk assessment^8^; and that only 18.5% of respondents recalled osteoporosis or fracture risk being discussed with specialists.^9^ The lack of attention to fracture risk is further compounded by the fact that people with PD under‐estimate their fracture risk.^10^

In the UK, according to the gold‐standard fracture liaison services (FLS), following a fragility fracture patients are expected to have a bone health assessment to determine if they are: low risk, and therefore offered life‐style advice to minimize their risk of future fractures; or high risk, and are therefore offered osteoporosis drug treatment.

Since bone health is often overlooked, we assessed the extent to which these treatment guidelines were followed in a population with parkinsonism following a fragility fracture. We sought to: (1) understand the extent to which bone protective medications and calcium/vitamin D were prescribed within 16 and 48‐weeks of a fragility fracture; (2) identify demographic and clinical factors which predict prescriptions being made using logistic regression.

## Methods

### Data Source

For this retrospective observational study, we used routinely‐collected general practice (GP) data within the Clinical Practice Research Datalink (CPRD) GOLD together with Office for National Statistics (ONS) mortality data. In 2013, CPRD contained 4.4 million active patients, representing 6.9% of the UK population from a network of 674 GP practices, with an additional 6.9 million inactive patients which includes those who have died or are no longer contributing data.^11^ Medical events are recorded within the clinical and referral files using Read codes, and prescription medications are recorded using British National Formulary (BNF) coding. CPRD therefore offers a unique opportunity to study the osteoporosis treatment gap in a large representative cohort of individuals with parkinsonism who have experienced at least one fragility fracture across the UK, where the treatment gap is defined as those who require treatment but are not receiving it within the given population.

### Data Extraction

All individuals with a Read code for parkinsonism (excluding drug‐induced parkinsonism, Table A1) in the CPRD clinical and/or referral files who contributed data between January 1, 2010 and December, 31 2019 were extracted. Patients were included if they received a diagnosis of parkinsonism prior to or during the study period. Patients were excluded if: they were censored out of CPRD prior to the study period or the censor date was prior to the first instance of a Read code for parkinsonism, since this suggested a likely data error; they had a Read code for parkinsonism prior to age 35; or there was no available data on primary care prescriptions (CPRD therapy data). This is because Read codes prior to age 35 potentially relate to a coding error, and the lack of prescription data indicates that there is likely to be further missing data.

### Study Population

The index date was the date of the first fragility fracture which occurred in the study period (January 1, 2010–December 31, 2019) for patients with prevalent parkinsonism, registered at a practice contributing data deemed to be of research quality, which is determined by the recording and consistency of variables including practice registration date, transfer out date and date of birth.^12^ Patients were followed‐up until they received a prescription for bone protective medication up to a maximum of 48‐weeks; or until they were censored, whichever came first. This was repeated considering prescriptions for vitamin D and/or calcium supplementation. The censor date was defined as the earliest of: date of death; the last day of data collection at a given GP practice; the date of transfer to another practice without CPRD data‐linkage, or the study end date. If patients received a prescription prior to their fragility fracture, they were not followed up beyond the index date.

In this study, fragility fracture sites considered were hip, vertebrae, wrist/distal radius, humerus, rib, pelvis and unspecified osteoporotic fractures. We followed the methods described by Watson et al 2017 to form a list of Read codes indicating the presence of a fragility fracture (fracture codes) (Table A2).^13^ The list was reviewed by two independent clinicians and any discrepancies were agreed by a third clinician. These Read codes were then used to identify patients who experienced at least one fragility fracture during the study period.

Fragility fractures were grouped into fracture events, and the initial fracture event within the study period was retained in the analysis as the index fracture event. Exact duplicates were removed from the dataset. To avoid double counting of fracture events, if two or more fracture codes related to the same fracture site or if an osteoporotic fracture at an unspecified site occurred less than 31 days apart, they were considered to be the same fracture event. Fracture events could consist of more than one specified fracture site if they occurred less than 31 days apart, and information about each fracture present at the event was retained.

### Prescription Data

Prescription data relating to either bone protective medications, or vitamin D and/or calcium supplementation were extracted from the CPRD therapy file using the BNF chapters 060602 (bone protective medications), 090604 (vitamin D supplementation), and 09050101 (calcium supplementation). Additionally, the medication descriptions were searched for relevant terms (Table A3), as some drugs were listed in other BNF chapters. Medications were cross‐referenced with the NICE guidelines, and drugs which were not licensed for use in osteoporosis, calcium or vitamin D deficiency, were removed. Pamidronate was excluded from this list as it is typically used in the context of malignancy. All prescriptions from the CPRD therapy file refer to drugs prescribed/dispensed in primary care for patients in all settings (own home, assisted living, care home etc).

Using index fragility fracture date, we determined whether patients were prescribed bone protective medications or vitamin D and/or calcium supplements before, or whether treatment was initiated after the fracture event. Patients were considered on treatment at the fracture event if prescription for oral bisphosphonates, strontium ranelate or teriparatide were received up to 3 months before; or if denosumab or zoledronate were documented in their medical records up to 9 or 18 months before respectively. These treatment windows were chosen using the standard dosing regimen for denosumab and zoledronate allowing for potential delays in treatment administration. Based on the UK Fracture Liaison Service gold‐standard, stating that appropriate osteoporosis drug treatment should be commenced within 16‐weeks of a fragility fracture,^14^ we analyzed 16‐week time intervals following the fracture event to determine adherence to this standard.

### Incomplete Follow‐Up

We considered, within each 16 week time interval, how many patients remained within the study and therefore could have possibly received a prescription. Patients no longer remained within the study if they were censored out (eg, death, transfer out) prior to receiving a prescription. This was considered separately for the bone protective and supplementation prescription data. The patients remaining within each time interval are reported and used as the denominator to calculate the proportion on treatment within each time window. The total number of patients censored from both the bone protective medications and supplementation data, and the reasons for censorship, are also described.

Bone protective medication and supplementation prescription data were considered separately, since these may not be initiated concurrently, potentially resulting in a patient being censored after prescription of only one medication type.

### Statistical Analysis

Stata 18 was used for the analysis.^15^ Data are described using mean/standard deviation, and the incidence of fractures is reported per 1000 person‐years with 95% confidence intervals. Logistic regression was used to obtain the prescription probability within a clinically meaningful time window and to test potential predictors of receiving prescriptions including: gender, age (at the mid‐point of the year of birth to the index fragility fracture date), PD duration at the time of fracture (calculated from the date at which the first diagnostic code for parkinsonism was applied), fracture site, Cambridge Multimorbidity Score (CMS) and whether the patient was resident in a care home at the time of fracture. The CMS quantifies multimorbidity using a count of 37 conditions based on diagnostic and prescription codes.^16^ Minimally adjusted models were determined using directed acyclic graphs (Figure A1). Two sensitivity analyses were conducted, restricting the analysis to: (i) patients with an initial diagnosis of PD; (ii) patients with 48‐weeks of data before and after the index fracture to test whether data availability affected any observed relationships.

## Results

We identified 21,792 people with parkinsonism, of whom 211 were excluded (n = 1 and n = 136 due to censoring before the study period or first parkinsonism code respectively; n = 33 due to missing prescription data; n = 41 due to age Fig. 1). The mean duration of available data was 3.1 years (SD = 2.5 years, range 1 day to 10.0 years). The mean age of patients at entry to the cohort was 74.7 years (SD = 9.9).

**Figure 1:**
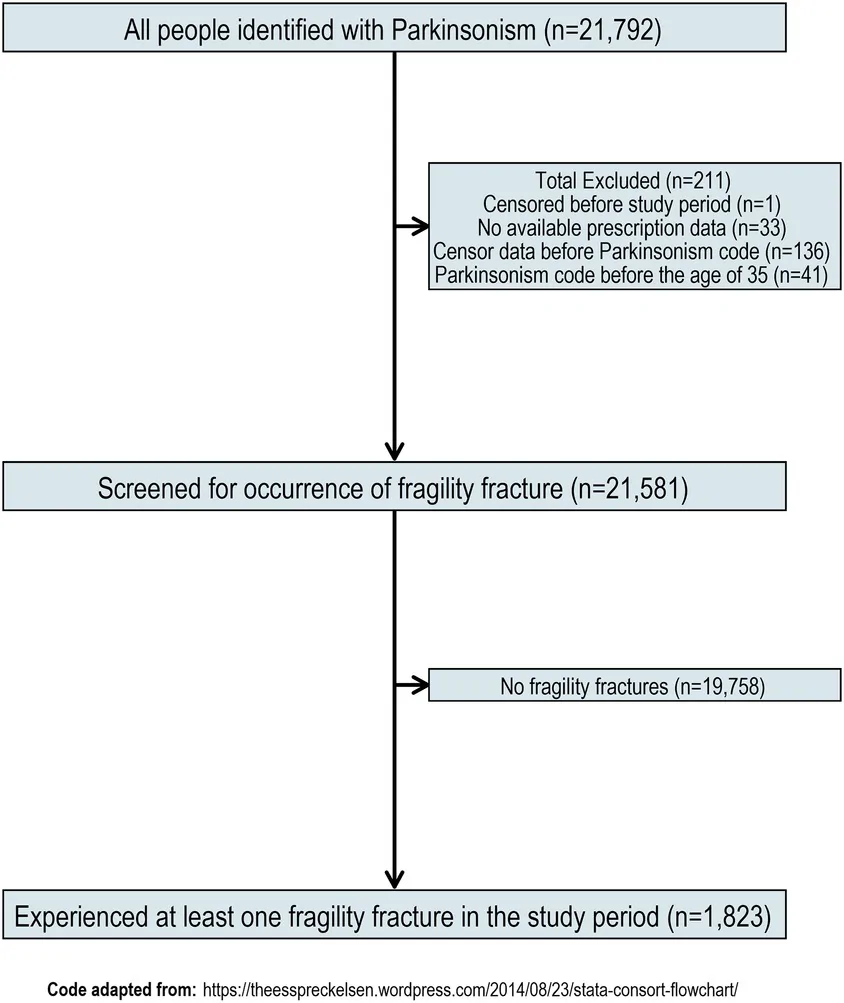
Flowchart showing the inclusion/exclusion of people from study population.

There were 1823 (8.4%) people with parkinsonism who had at least one fragility fracture during the study period. The incidence rate of fragility fractures was 27.3 per 1000 person‐years (95% CI = 26.1–28.6). The mean age at the time of fracture was 78.9 (SD = 7.79) years. Of those who experienced a fracture within the study period, hip fractures were the most common, experienced by 813 people (44.6%), followed by vertebral fractures in 255 people (14.0%). Within a 31‐day period, which we assumed to relate to the same fracture event, only 29 (1.6%) people experienced two different fractures and 1 (0.1%) experienced three (Table 1).

**TABLE 1:** Demographic characteristics of the parkinsonian cohort identified in clinical practice research datalink (CPRD) 2010–2019, and the fracture subgroup

	Full cohort	Fracture subgroup
	Mean (SD) or N (%)	Mean (SD) or N (%)
N	21,581	1823
Age upon entry to cohort^a^	74.7 (9.8)	76.5 (8.1)
Sex		
Male	12,942 (60.0%)	751 (41.2%)
Female	8639 (40.0%)	1072 (58.8%)
PD duration (years)	1.4 (3.5)	1.9 (3.7)
Diagnosis		
Parkinson's disease	17,897 (82.9%)	1521 (83.4%)
Dementia with Lewy bodies	1716 (8.0%)	122 (6.7%)
Vascular Parkinsonism	929 (4.3%)	78 (4.3%)
Progressive supranuclear palsy	374 (1.7%)	37 (2.0%)
Parkinson's disease dementia	206 (1.0%)	15 (0.8%)
Multiple system atrophy	166 (0.8%)	10 (0.5%)
Corticobasal degeneration	65 (0.3%)	4 (0.2%)
Parkinsonism	12 (0.1%)	0
Other^b^	216 (1.0%)	36 (2.0%)
Years of available CPRD data	3.1 (2.5)	4.3 (2.6)
Fragility fracture present in study period		
None	19,758 (91.6%)	–
One or more	1823 (8.4%)	–
How many individual fractures occurred in this first event?		
1 Fracture	–	1793 (98.4%)
2 Fractures	–	29 (1.6%)
3 Fractures	–	1 (0.1%)
Age at fracture	–	78.9 (7.8)
Fracture site^c^		
Hip	–	813 (44.6%)
Vertebral	–	255 (14.0%)
Wrist	–	125 (6.9%)
Humerus	–	197 (10.8%)
Rib	–	115 (6.3%)
Pelvis	–	78 (4.3%)
Unspecified	–	269 (14.8%)

^a^ This is age on January 1, 2010 for those diagnosed with Parkinson's before the study period began, or age on the day of Parkinson's diagnosis if diagnosed during the study period.

^b^ This includes the Read codes for paralysis agitans, Parkinson's orthostatic hypotension and cerebral degeneration PD, all of which indicate Parkinsonism.

^c^ These frequencies reflect the number of participants whom experienced a fracture at each site rather than the number of fractures.

### Prescriptions for Bone Protective Medications and Supplementation at Indexed Fracture Event

Of those with a fragility fracture event, 225 people (12.3%) were on any bone protective medication prior to their fracture. A further 486 people were started on bone protective medication within 48 weeks following their fracture, therefore cumulatively 52.9% were in receipt of a prescription within 48 weeks (Fig. 2A and Table A4). Four hundred and forty two (24.2%) were prescribed vitamin D and/or calcium supplementation prior to their fracture, and a further 651 people were prescribed supplementation up to 48 weeks after their fracture, therefore cumulatively 73.2% were in receipt of a prescription within 48 weeks (Fig. 2B and Table A5).

**Figure 2:**
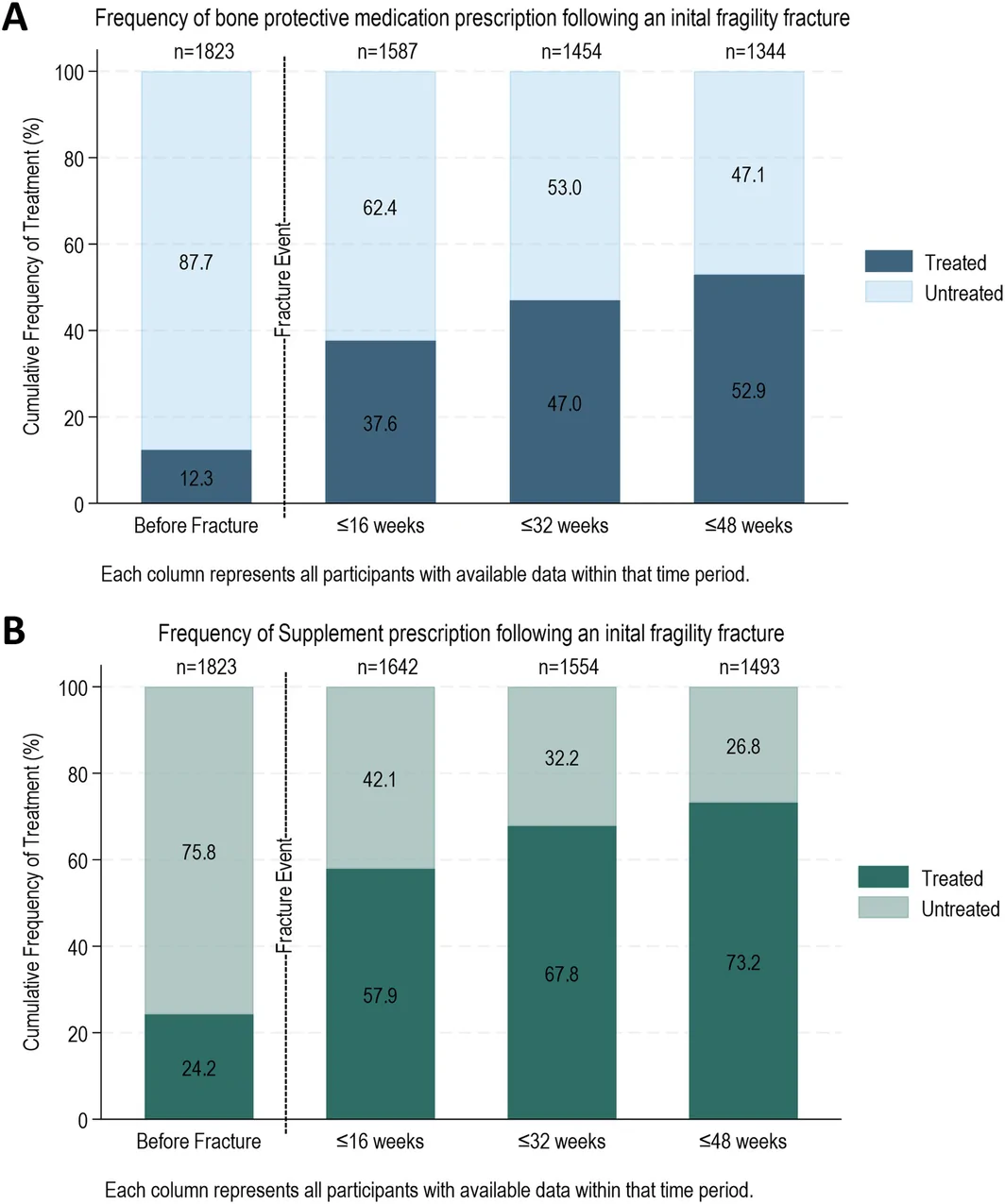
Stacked bar charts illustrating the prescription of (A) bone protective medication (B) vitamin D/calcium supplementation prior to and following a fragility fracture.

### Incomplete Follow‐Up

A total of 479 patients were censored from the bone protective medication data within 48 weeks of a fragility fracture, due to 123 deaths, 53 patients reaching the last point of data collection from the practice, 207 transferring to a GP practice without CPRD linkage, and 96 reaching the end of the study period prior to a prescription being issued (Figure A2). Of the 1598, who were not prescribed bone protective medication prior to their fragility fracture, the mean follow‐up time was 1.2 years (SD = 1.6 years, range 0 days to 9.7 years).

With respect to vitamin D and/or calcium prescriptions, a total of 330 patients were censored within 48 weeks of a fragility fracture, due to 92 deaths, 37 patients reaching the last point of data collection from the practice, 149 transferring to a GP practice without CPRD linkage, and 52 reaching the end of the study period prior to a prescription being issued (Figure A3). Of the 1381, who were not prescribed vitamin D and/or calcium prior to their fragility fracture, the mean follow‐up time was 0.9 years (SD = 1.5 years, range 0 days to 9.7 years).

### Predictors of Bone Protective Prescriptions

We explored demographic factors and fracture site as predictors of bone protective prescriptions being issued (Table 2). Being female (OR 2.06 [95% CI (1.69–2.51)], *p* < 0.001) and being aged 75–79 (OR 1.82 [95% CI (1.40–2.37)], *p* < 0.001), 80–84 (OR 1.67 [95% CI (1.28–2.17)], *p* < 0.001) or over 85 (OR 1.63 [95% CI (1.24–2.16)], *p* = 0.001), increased the odds of bone protective medication prescription. Residing in a care home reduced the odds of bone protective medication prescription (OR 0.64 [95% CI (0.43–0.96)], *p* = 0.03). Sustaining a vertebral fracture (OR 1.84 [95% CI (1.38–2.45)], *p* < 0.001), or having unspecified fragility fracture recorded (OR 1.76 [95% CI (1.33–2.33)], *p* < 0.001), when compared to sustaining a hip fracture, also increased the odds of bone protective medication prescription, whereas sustaining a rib fracture (OR 0.28 [95% CI (0.16–0.49)], *p* < 0.001) reduced the odds.

**TABLE 2:** Unadjusted logistic regression models to look at factors predicting bone protective medication prescriptions

	Unadjusted odds ratio (95% CI)	N	*p*‐value
Gender			
Male	ref	1823	
Female	2.06 (1.69–2.51)		<0.001
Age			
35–74	ref	1823	
74–79	1.82 (1.40–2.37)		<0.001
80–84	1.67 (1.28–2.17)		<0.001
85+	1.63 (1.24–2.16)		0.001
PD duration			
Less than 1 year	ref	1823	
1–2.4 years	0.78 (0.59–1.03)		0.08
2.5–4 years	0.80 (0.61–1.05)		0.11
5–9 years	0.79 (0.59–1.05)		0.10
10+ years	0.76 (0.54–1.07)		0.12
Fracture type			
Hip	ref	1823	
Vertebral	1.84 (1.38–2.45)		<0.001
Wrist	0.68 (0.44–1.05)		0.08
Humerus	0.80 (0.57–1.12)		0.19
Rib	0.28 (0.16–0.49)		<0.001
Pelvis	1.14 (0.71–1.85)		0.58
Unspecified	1.76 (1.33–2.33)		<0.001
Multiple Fractures	1.30 (0.62–2.71)		0.49
Cambridge multimorbidity score			
0/1	ref	1823	
2	0.82 (0.51–1.33)		0.42
3	0.83 (0.53–1.31)		0.43
4	0.91 (0.58–1.43)		0.68
5	0.93 (0.59–1.46)		0.74
6	0.95 (0.59–1.52)		0.83
7+	0.88 (0.56–1.38)		0.57
Care home residence			
Not living in care home	ref	1823	
Living in care home	0.64 (0.43–0.96)		0.03

When adjusting for age and gender, vertebral fractures (OR 2.06 [95% CI (1.53–2.77)], *p* < 0.001) had stronger association with receiving a prescription whereas unspecified fracture (OR 1.57 [95% CI (1.17–2.10)], *p* < 0.001), and rib fractures (OR 0.34 [95% CI (0.19–0.60)], *p* < 0.001) both had a slightly attenuated relationship with receiving a prescription for bone protective medication versus hip fracture. When adjusting for age, multi‐morbidity (CMS) and gender, living in a care home (OR 0.58 [95% CI (0.38–0.88)], *p* = 0.01) had a stronger association of decreasing the odds of receiving a prescription than in the univariate model. Multimorbidity still did not change the odds of receiving a prescription after adjusting for age (Table 3). The direction of these associations was not altered when restricting the analysis to those with an initial diagnosis of PD or those with 48 weeks of data before and after the index fracture, although it did weaken the association between prescriptions and care home residency (Table A6 and A7).

**TABLE 3:** Minimally adjusted logistic regression models to look at factors predicting bone protective medication prescriptions

	Odds ratio (95% CI)	N	*p*‐value
Fracture type (unadjusted)			
Hip	ref	1823	
Vertebral	1.84 (1.38–2.45)		<0.001
Wrist	0.68 (0.44–1.05)		0.08
Humerus	0.80 (0.57–1.12)		0.19
Rib	0.28 (0.16–0.49)		<0.001
Pelvis	1.14 (0.71–1.85)		0.58
Unspecified	1.76 (1.33–2.33)		<0.001
Multiple Fractures	1.30 (0.62–2.71)		0.49
Fracture type adjusted for age			
Hip	ref	1823	
Vertebral	1.98 (1.48–2.65)		<0.001
Wrist	0.75 (0.49–1.17)		0.21
Humerus	0.82 (0.58–1.16)		0.26
Rib	0.31 (0.18–0.55)		<0.001
Pelvis	1.15 (0.71–1.86)		0.57
Unspecified	1.84 (1.39–2.44)		<0.001
Multiple fractures	1.29 (0.62–2.71)		0.50
Fracture type adjusted for age and gender			
Hip	ref	1823	
Vertebral	2.06 (1.53–2.77)		<0.001
Wrist	0.60 (0.38–0.93)		0.02
Humerus	0.72 (0.50–1.02)		0.06
Rib	0.34 (0.19–0.60)		<0.001
Pelvis	0.99 (0.61–1.61)		0.96
Unspecified	1.57 (1.17–2.10)		<0.001
Multiple fractures	1.23 (0.58–2.61)		0.59
Cambridge multimorbidity score (unadjusted)			
0/1	ref	1823	
2	0.82 (0.51–1.33)		0.42
3	0.83 (0.53–1.31)		0.43
4	0.91 (0.58–1.43)		0.68
5	0.93 (0.59–1.46)		0.74
6	0.95 (0.59–1.52)		0.83
7+	0.88 (0.56–1.38)		0.57
Cambridge multimorbidity score adjusted for age			
0/1	ref	1823	
2	0.77 (0.47–1.25)		0.29
3	0.76 (0.48–1.21)		0.25
4	0.81 (0.51–1.28)		0.37
5	0.81 (0.51–1.28)		0.36
6	0.82 (0.51–1.32)		0.41
7+	0.77 (0.49–1.21)		0.26
Care home residency (unadjusted)			
No	ref	1823	
Yes	0.64 (0.43–0.96)		0.03
Care home residency adjusted for age			
No	ref	1823	
Yes	0.58 (0.39–0.88)		0.01
Care home residency adjusted for age and gender			
No	ref	1823	
Yes	0.58 (0.38–0.88)		0.01
Care home residency adjusted for age, gender and CMS			
No	ref	1823	
Yes	0.58 (0.38–0.88)		0.01

## Discussion

Despite a significantly increased risk of sustaining fragility fractures, bone‐health and osteoporosis management is often overlooked when caring for people with parkinsonism. To understand the extent to which osteoporosis is treated in line with national guidelines, we investigated whether either bone protective medications, vitamin D and/or calcium supplements were prescribed. This was based on primary care records before and after people experienced fragility fractures. We identified treatment gaps and investigated potential predictors of prescriptions being made within 48 weeks following a fracture.

We identified a fracture rate of 27.3 per 1000 person years which aligns with the current literature.^6^, ^17^ Consistent with our previous findings^18^ and with the results of the Parkinson's UK National Audit,^9^ we identified a substantial treatment gap. Only 38% of this real‐world population who sustained a fragility fracture were receiving treatment within the recommended 16 week period. Increasing the treatment window to 48 weeks post fracture only increased this proportion to 53%. More people were prescribed vitamin D and/or calcium supplementation within 16 (58%) and 48 weeks (73%). Being female, aged >75 years and having sustained either a vertebral or unspecified fragility fracture increased the odds of receiving a prescription for bone protective medications. Conversely, people with parkinsonism who had sustained rib fracture(s), or were residing in a care home were less likely to be prescribed bone protective medication. The direction of these associations was maintained when restricting the analysis to those with: more complete data (Table A7); or an initial diagnosis of PD (Table A6). Due to initial diagnostic uncertainty, it is possible that some PD diagnoses may subsequently have been revised to an atypical parkinsonism.^19^

Whilst demonstrating this striking and stark treatment gap, we have considered additional factors which may have impacted this finding. Using CPRD data it is possible to see which patients were prescribed bone protective medication in primary care. There is insufficient data to reliably determine whether bone health was considered and then no treatment was initiated (appropriately), adherence to prescribed treatments, or whether patients had received intravenous zoledronate via secondary care. There are several reasons why osteoporosis treatment may have been deemed inappropriate despite a patient sustaining a fragility fracture, including: concerns over polypharmacy; upper gastrointestinal disorders; renal impairment; patient preferences; bisphosphonate treatment holidays; life‐expectancy; concerns over side‐effects; or fracture risk assessment concluding treatment wasn’t warranted.^20^, ^21^ These legitimate reasons why treatments are sometimes not initiated will contribute to an over‐estimation of the reported treatment gap. We might hypothesize that individuals with greater co‐morbidity burden are less likely to receive a prescription, but this was not evident in our data.

It is unclear to what extent the inability to capture treatment with zoledronate affects the treatment gap during the study period (2010–2019). Data from the National Hip Fracture Database (NHFD) indicates that in 2020 only 9% of patients were started on zoledronate following a hip fracture,^22^, ^23^ and in 2021 NOGG guidance was updated to recommend zoledronate as a first‐line treatment.^21^, ^24^ Prior to 2021, the recommendation was to use zoledronate if first‐line treatments, alendronate and risedronate, were not tolerated or contraindicated.^25^, ^26^ However, given that people with PD commonly experience difficulty swallowing,^27^ perhaps oral bisphosphonates were deemed inappropriate and zoledronate was considered a suitable alternative in some instances. Therefore, the inability to capture zoledronate may contribute to an over‐estimation of the identified treatment gap, however this gap is unlikely to be fully explained by this.

We excluded missing data from the treatment gap analysis. However, it is also important to consider those who did not receive a prescription before the last collection date for the practice, the end of the study period or who transferred to a practice without CPRD follow‐up. It is possible that these patients were later provided osteoporosis treatment. Amongst patients who died within 48 weeks and did not receive bone protective medication or supplementation, this may indicate a clinical decision that treatment was unlikely to be beneficial due to limited life expectancy.

When exploring demographic factors as predictors for bone protective medication prescription, it was unsurprising that being female compared to male increased the odds of prescriptions (OR 2.06 [95% CI (1.69–2.51)], *p*<0.001), given the known gender disparities in the treatment of osteoporosis. Recent work has demonstrated that in vertebral fracture cohorts the odds ratio for treatment was 1.82 favoring women^28^ and, at hospital discharge following a hip fracture, only 2.7% (n = 3/110) of men compared to 27% (n = 60/253) of women were on anti‐resorptive treatment in a single center study in the United States.^29^

Fracture type also affected the odds of receiving a prescription for bone protective medications. Vertebral fractures (OR 2.06 [1.53–2.77], *p* < 0.001) and unspecified osteoporotic fractures (OR 1.57 [1.17–2.10], *p* < 0.001) when compared to hip fracture increased the odds, whereas rib fractures (OR 0.34 [0.19–0.60], *p* < 0.001) when compared to hip fractures decreased the odds of prescription. We hypothesize that this could be explained by the different inpatient treatment received, although this cannot be addressed using CPRD data. In the UK, rib fractures, unlike hip fractures, are more variably managed in different inpatient locations besides geriatrics, including orthopedics and general surgery^30^ where there may be less familiarity with the use of osteoporosis treatments potentially leading to a discrepancy in the recommendation for bone protective medications. The finding that those with vertebral versus hip fracture have increased odds of receiving a prescription reflects findings from the US that following a vertebral fracture there is the largest increase in osteoporosis diagnosis and treatment rates compared to other fracture types.^31^

Care home residency also reduced the odds of prescription after adjustment for age, gender and number of comorbidities (OR 0.58 [0.38–0.88], *p* = 0.01). However, the evidence for this was weakened when restricting the analysis to those with an initial diagnosis of PD or to those with 48 weeks of data before and after the index fracture. This is most likely explained by the small number of patients who were resident in a care home in these sub‐groups (Table A6 and A7) therefore widening the confidence interval. This result is consistent with findings from US care home residents with dementia and high fracture risk, where only 11.6% received fracture prevention medications.^32^ The authors also highlighted a potential lack of awareness about the appropriateness of treatment, as 10% of those receiving hospice care and 11% of individuals who were not dependent for activities of daily living were receiving medication for fracture prevention reflecting over and under‐treatment respectively.^32^ Whilst the appropriateness of treatment could not be assessed in our study, it is important to consider that treatment for osteoporosis should be considered on a case‐by‐case basis accounting for several patient factors regardless of whether the patient resides in a care home.

## Strengths and Limitations

This study is strengthened by the ability to assess the management of osteoporosis in a large, representative cohort of people with Parkinsonism across the United Kingdom. By using CPRD data it is possible to include previously under‐represented groups such as those who are older, with more advanced parkinsonism, care home residents, as well as people across the spectrum of parkinsonian disorders.^33^ This is of particular importance given that people with atypical parkinsonism have greater fracture risk, alongside an osteoporosis treatment gap,^34^, ^35^ and there is often diagnostic uncertainty.^19^

It was beyond the scope of this paper to ascertain whether patients were adherent to prescribed treatment or whether health care professional follow‐up was provided to maximize adherence.^14^ Adherence to osteoporosis treatment is a well‐documented issue; persistence to oral bisphosphonates amongst post‐menopausal women is reported to be only 28% at 12 months.^36^ Therefore, despite prescriptions being made it is likely that many patients were still without treatment following a fragility fracture.

When determining if patients were on treatment prior to fracture, for oral bisphosphonates patients were considered “on‐treatment” if they received a prescription in the 3 months prior to their fracture. However, this could mis‐classify participants if they were on treatment holiday after previously being prescribed oral bisphosphonates. It was also likely that patients being given zoledronate are mis‐categorized as “untreated” as it is not administered in the primary care setting and is therefore not reliably recorded within the CPRD data. Furthermore, patients could have been misclassified as “not taking supplementation” if they are using over‐the‐counter vitamin D and/or calcium. However over‐the‐counter formulations may be sub‐standard in comparison to licensed medical products.^37^ All of which could contribute to the observed treatment gaps.

As previously discussed, it is also possible that treatment options were considered and deemed inappropriate or declined by patients; however, this information is not routinely coded in CPRD data. It is also not possible using CPRD data to determine the circumstances surrounding the fracture. Whilst we included fracture sites typically associated with fragility fractures, it is possible that some of these fractures occurred from a trauma greater than a fall from standing height and are also contributing to an over‐estimation of the treatment gap as they are not indicative of osteoporosis.

Additionally, this population includes all people with parkinsonism who experienced a fracture between 2010 and 2019; in this study we consider the treatment decisions made after only the initial fracture which occurred within this period. However, it is possible that patients had multiple fracture events within this period or had fractures prior to 1st January 2010. Furthermore, the data available to us for analysis pre‐dates the updated NOGG guidance which mentions Parkinson's as an osteoporosis risk factor,^21^ and our guidance on managing bone‐health in parkinsonism.^18^, ^38^ However, the treatment gap identified remains consistent with our 2025 findings that less than one third of people with parkinsonism are treated in‐line with the NOGG guidelines.^18^

There remains a significant osteoporosis treatment gap within the parkinsonian population. Men with parkinsonism, those living in a care home, and those who sustain rib fractures are at increased odds of not receiving a prescription for bone protective medication following a fracture. Addressing this gap might reasonably be expected to prevent future fragility fractures and the associated serious and negative sequalae in this vulnerable and at‐risk population.

## Author Roles

(1) Research project: A. Conception, B. Organization, C. Execution, (2) Statistical analysis: A. Design, B. Execution, C. Review and critique; (3) Manuscript preparation: A. Writing of the first draft, B. Review and critique.

K.C.N.: 1A, 1B, 1C, 2A, 2B, 3A.

E.J.H.: 1A, 1B, 1C, 2A, 2C, 3B.

E.T.: 1A, 1B, 1C, 2A, 2C, 3B.

## Disclosures

**Ethical Compliance Statement:** This study is based in part on data from the Clinical Practice Research Datalink obtained under license from the UK Medicines and Healthcare products Regulatory Agency (protocol number 20_000060). However, the interpretation and conclusions contained in this report are those of the author's alone. The authors confirm that the approval of an institutional review board and patient consent were not required for this work. We confirm that we have read the Journal's position on issues involved in ethical publication and affirm that this work is consistent with those guidelines.

**Funding Sources and Conflict of Interest:** This study was funded by the Gatsby Foundation (GAT3676). EJH is HEFCE funded for her academic work. ET is funded by a National Institute for Health and Care Research Academic Clinical Lectureship. The authors declare that there are no conflicts of interest relevant to this work.

**Financial Disclosures for the Previous 12 Months:** EJH is HEFCE funded by University of Bristol for her academic work and has received research funding from the NIHR, the British Geriatrics Society, the Gatsby Foundation, the Alzheimer's Society, Royal Osteoporosis Society, the Dunhill Society, and Parkinson's UK. She has received travel support, honoraria, and/or sat on advisory boards for Kyowa Kirin, AbbVie, Luye, the CME institute, Ever, Simbec Orion, the Neurology Academy, and Bial. ET is funded by a National Institute for Health and Care Research Academic Clinical Lectureship. KCN is funded by the University of Bristol.

## Figures and Tables

**Figure A1:**
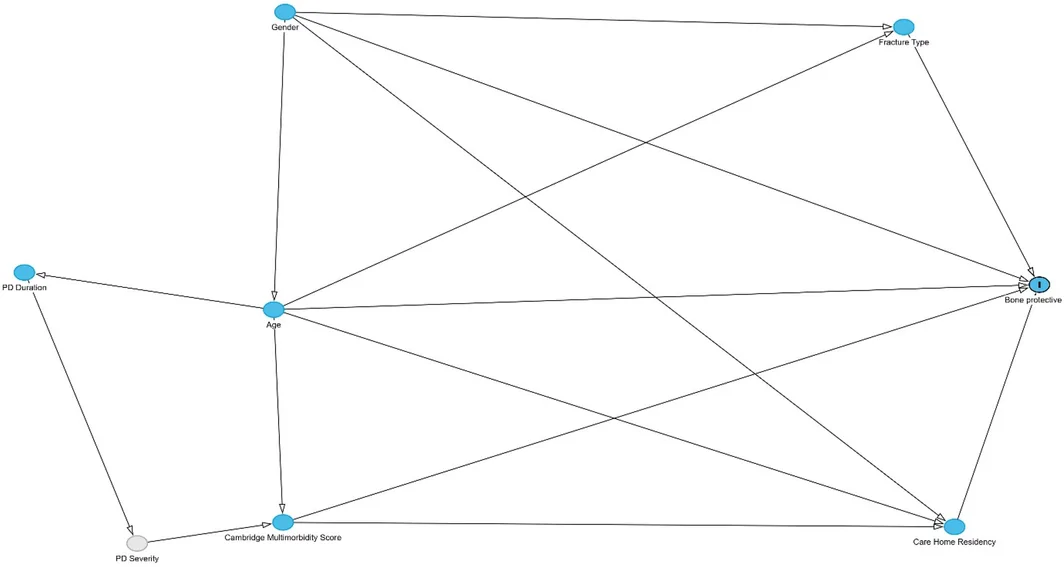
Directed acyclic graphs for the minimally adjusted logistic regressions. This directed acyclic graph was developed using the DAGitty software^1^ to visually represent the causal and non‐causal associations that informed the minimally adjusted logistic regression models. ^1^1 Textor J, van der Zander B, Gilthorpe MS, Liskiewicz M, Ellison GT. Robust causal inference using directed acyclic graphs: the R package “dagitty.” Int J Epidemiol. 2016;45(6):1887–94.

**Figure A2:**
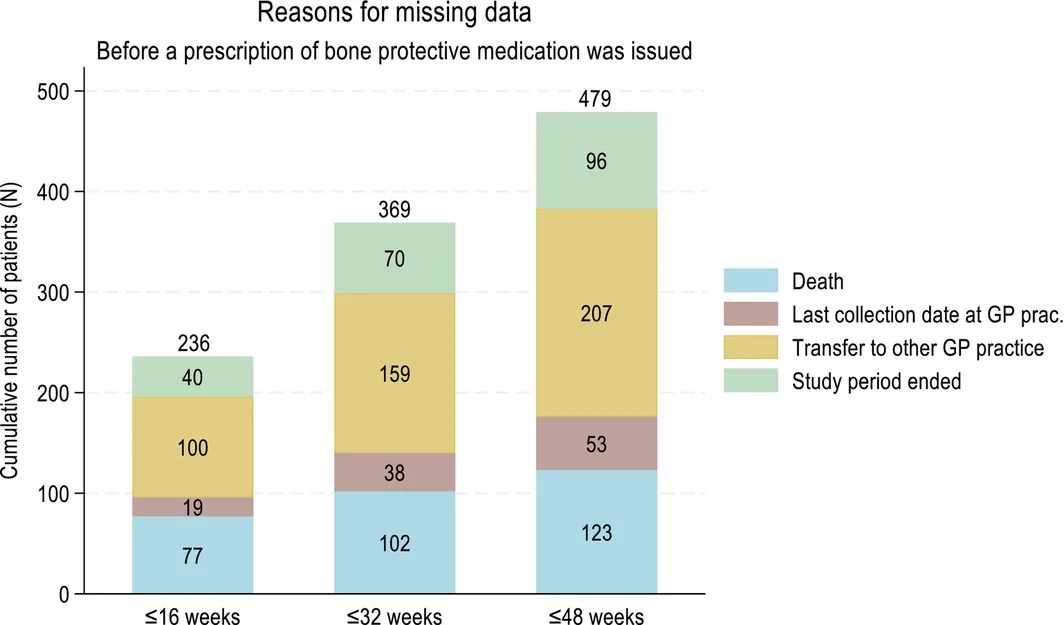
Reasons for incomplete follow‐up–bone protective medications.

**Figure A3:**
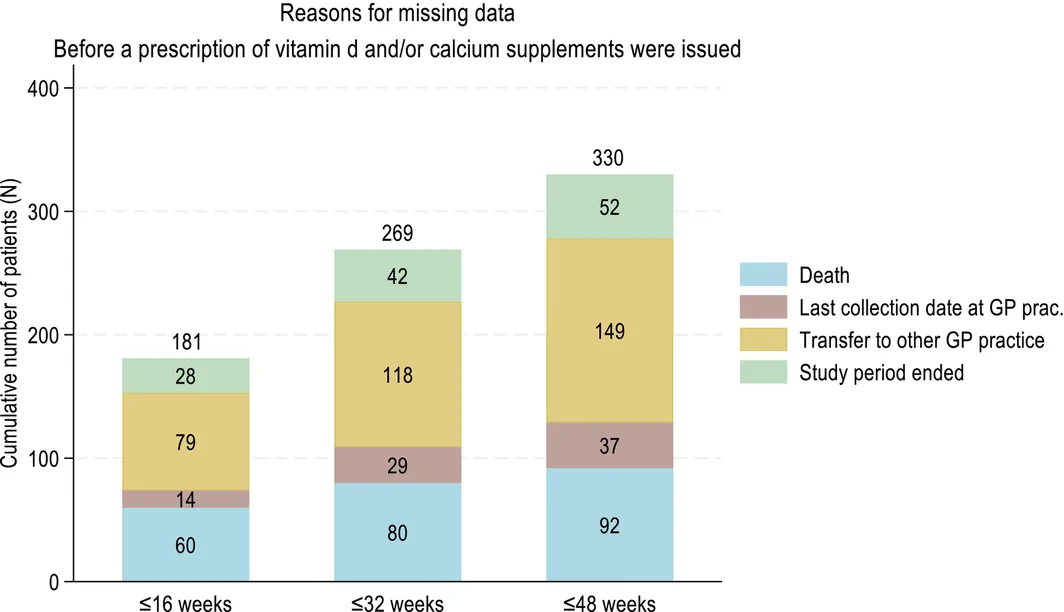
Reasons for incomplete follow‐up–vitamin D and/or calcium supplements.

## Data Availability

This study is based in part on data from the Clinical Practice Research Datalink obtained under license from the UK Medicines and Healthcare products Regulatory Agency. However, the interpretation and conclusions contained in this report are those of the author's alone.

## Appendix A

**TABLE A1:** Included parkinsonism read codes

Description (as listed in CPRD medical.txt file)	Medcode	Read code
Parkinson's disease	4321	F12.00
Parkinsonism with OH	8956	F130300
Dementia in Parkinson's disease	9509	Eu02300
Parkinson's disease NOS	14,912	F12z.00
Parkinsonism in diseases classified elsewhere	86,062	Fyu2200
Paralysis agitans	1691	F120.00
Vascular parkinsonsim	100,128	F124.00
Lewy body disease	7572	F116.00
[X]Lewy body dementia	26,270	Eu02500
Multiple system atrophy	22,454	F174.00
Shy‐Drager syndrome	35,839	F130500
Progressive supranuclear palsy	9385	F24y000
Progressive supranuclear ophthalmoplegia	40,553	F130400
Steele‐Richardson Oszewski syndrome	93,910	F24y012
Steele Richardson Oszewski syn	49,034	F24y011
Steele‐Richardson‐Oszewski syndrome	7037	F24y200
Supranucelar paralysis	29,515	F36y000
Corticobasal degeneration	107,650	F11y200
Cerebral degeneration in Parkinson's disease	96,860	F11x900

**TABLE A2:** Included fragility fracture read codes.

Description (as listed in CPRD medical.txt file)	Medcode	Read code
Hip		
Hip fracture	1994	S30.11
Fracture of neck of femur	2225	S30.00
Closed fracture of proximal femur, pertrochanteric	5301	S302.00
Prmy open red+int fxn prox femoral # + screw/nail+plate device	5742	7K1D000
Closed reduction of fracture of hip	6660	7K1L400
Closed fracture of femur, intertrochanteric	8648	S302400
Cls red+int fxn proximal femoral # + screw/nail device alone	8719	7K1J000
DHS‐Dynamic hip screw primary fixation of neck of femur	9792	7K1D01E
Hip fracture NOS	10,570	S30y.11
Dynamic hip screw primary fixation of neck of femur	12,544	7K1D01F
Cls # prox femur, subcapital, Garden grade unspec.	17,019	S300500
H/O: hip fracture	17,936	14G7.00
Closed fracture of neck of femur NOS	18,273	S30y.00
Cls # proximal femur, trochanteric section, unspecified	19,117	S302000
Open fracture proximal femur, subcapital, Garden grade III	23,803	S301800
Closed fracture of unspecified proximal femur	24,276	S30w.00
Prim open reduct # neck femur & op fix‐Richards screw	24,493	7K1D01A
Pertrochanteric fracture	28,965	S304.00
Closed fracture proximal femur, subtrochanteric	29,145	S302200
Prmy open red+int fxn prox femoral # + screw/nail device alone	33,624	7K1D600
Closed fracture proximal femur, subcapital, Garden grade II	33,957	S300700
Closed fracture proximal femur, subcapital, Garden grade IV	34,078	S300900
Closed fracture proximal femur, subcapital, Garden grade I	34,351	S300600
Prmy open red+int fxn prox fem # + screw/nail+intramed device	34,764	7K1D700
Primary int fxn(no red) prox fem # + screw/nail device alone	35,004	7K1J500
Closed fracture head of femur	36,391	S300400
Closed fracture proximal femur, subcapital, Garden grade III	36,599	S300800
Open fracture of neck of femur NOS	38,054	S30z.00
Closed fracture proximal femur, transcervical	38,489	S300.00
Primary int fxn(no red) prox fem # + screw/nail+plate device	38,856	7K1J700
Open fracture proximal femur, subcapital, Garden grade unspec	38,878	S301500
Open fracture of femur, intertrochanteric	39,396	S303400
Cls # prox femur, intracapsular section, unspecified	39,984	S300000
Cl red intracaps fract neck femur fix − Smith‐Petersen nail	40,999	7K1J012
Primary int fxn(no red) prox fem # + scrw/nail+intramed device	44,594	7K1J600
Cls # of proximal femur, pertrochanteric section, NOS	44,735	S302z00
Closed fracture proximal femur, intertrochanteric, two part	45,141	S302100
Prim open reduct # neck femur & op fix − Neufield nail plate	46,258	7K1D018
Primary cls red+int fxn prox fem # + screw/nail+plate device	46,959	7K1JD00
Closed fracture proximal femur, other transcervical	49,209	S300y00
Opn # proximal femur, intracapsular section, unspecified	50,727	S301000
Cls # proximal femur, intertrochanteric, comminuted	51,216	S302300
Closed fracture, base of neck of femur	51,861	S300311
Open fracture proximal femur, subcapital, Garden grade IV	51,999	S301900
Closed fracture proximal femur, basicervical	52,194	S300300
Prim op red # nck femur and op fix‐Charnley compression screw	52,395	7K1D012
Cl red intracaps frac neck femur fix‐Garden cannulated screw	53,670	7K1J011
Prim cls rd + int fxn prox fem # + screw/nail+intramdulry device	54,819	7K1JC00
Primary cls red+int fxn prox fem # + screw/nail device alone	55,386	7K1JB00
Prim open red # neck femur and op fix‐McLaughlin nail plate	56,568	7K1D017
Cls red+int fxn prox femoral # + Richard's cannulat hip screw	57,514	7K1J013
Prim open reduct # neck femur and op fix‐Ross Brown nail	57,884	7K1D01B
Prim op red # nck femur and op fix‐Zickel intramed nail plate	57,889	7K1D01D
Open fracture of unspecified proximal femur	58,642	S30x.00
Prim open reduct # neck femur and op fix‐Blount nail plate	58,817	7K1D011
Open fracture proximal femur, subcapital, Garden grade I	60,885	S301600
Open fracture of proximal femur, pertrochanteric	61,733	S303.00
Closed fracture proximal femur, transcervical, NOS	62,966	S300z00
Prim open reduct # neck femur and op fix‐Pugh nail plate	65,536	7K1D019
Closed fracture proximal femur, midcervical section	65,690	S300200
Open fracture proximal femur, subcapital, Garden grade II	67,394	S301700
Open # of proximal femur, trochanteric section, unspecified	67,633	S303000
Closed fracture of femur, subcapital	68,229	S300y11
Open fracture proximal femur, other transcervical	68,668	S301y00
Primary external fixation (without reduction) prox femoral #	70,018	7K1K300
Open fracture of proximal femur, pertrochanteric, NOS	70,479	S303z00
Open fracture proximal femur, subtrochanteric	71,282	S303200
Open fracture of femur, subcapital	73,234	S301y11
Open fracture proximal femur, transcervical	73,981	S301.00
Closed fracture‐subluxation, hip joint	93,374	S4E2.00
Prim open reduct # neck femur and op fix‐Holt nail	94,714	7K1D014
Prim op red # nck femur and op fix‐Deyerle multiple hip pin	97,337	7K1D013
Open fracture proximal femur, intertrochanteric, comminuted	97,971	S303300
Open fracture base of neck of femur	100,771	S301311
Open fracture proximal femur, intertrochanteric, two part	101,567	S303100
Primary cls reduction+external fixation proximal femoral #	102,313	7K1K500
Prim open reduct # neck femur and op fix‐Jewett nail plate	105,352	7K1D015
Prim op red frac neck fem op fix us prox fem nail antirotatn	105,803	7K1DE00
Remanip intracap fract neck fem and fix using nail or screw	107,358	7K1Y000
Prim open reduct # neck femur and op fix‐Massie nail plate	107,932	7K1D016
Open fracture proximal femur, transcervical, NOS	112,712	S301z00
Open fracture proximal femur, midcervical section	112,836	S301200
Open fracture proximal femur, basicervical	113,426	S301300
Closed reduction of intracapsular # NOF internal fixat DHS	39,322	7K1Jd00
Vertebral fractures		
Collapse of vertebra NOS	2793	N331.12
Closed fracture lumbar vertebra	3888	S104.00
Collapse of vertebra due to osteoporosis NOS	4013	N331L00
Fracture of vertebra without spinal cord lesion	4409	S10.12
Fracture of thoracic vertebra	5381	S15.00
Collapse of lumbar vertebra due to osteoporosis	5841	N331J00
Closed fracture lumbar vertebra, wedge	8266	S104100
Collapse of thoracic vertebra	9319	N331F00
Fracture of lumbar vertebra	10,990	S10B000
Collapse of lumbar vertebra	11,543	N331G00
Other specified closed fracture thoracic vertebra	11,770	S102y00
Fracture of lumbar spine and pelvis	12,406	S10B.00
Osteoporosis + pathological fracture thoracic vertebrae	12,673	N331900
Closed fracture of unspecified cervical vertebra	15,613	S100000
Collapse of thoracic vertebra	15,837	N331011
Fatigue fracture of vertebra	16,895	N1y1.00
Collapse of cervical vertebra	17,008	N331E00
Osteoporosis + pathological fracture lumbar vertebrae	17,377	N331800
Collapse of thoracic vertebra due to osteoporosis	19,048	N331K00
H/O: vertebral fracture	19,235	14G8.00
Collapse of lumbar vertebra	23,686	N331111
Closed fracture of seventh cervical vertebra	24,672	S100700
Closed fracture thoracic vertebra	27,404	S102.00
Closed fracture of fifth cervical vertebra	27,575	S100500
Closed fracture of sixth cervical vertebra	27,654	S100600
Fracture of first cervical vertebra	28,133	S10A000
Closed fracture thoracic vertebra, wedge	28,524	S102100
C6 vertebra closed fracture without spinal cord lesion	33,503	S100611
C2 vertebra closed fracture without spinal cord lesion	33,967	S100211
Fracture of second cervical vertebra	34,403	S10A100
C5 vertebra closed fracture without spinal cord lesion	34,873	S100511
C7 vertebra closed fracture without spinal cord lesion	38,053	S100711
Collapsed vertebra NOS	38,728	N331D00
Closed fracture thoracic vertebra not otherwise specified	41,138	S102z00
Closed fracture of fourth cervical vertebra	41,548	S100400
C1 vertebra closed fracture–no spinal cord lesion	42,561	S100111
Osteoporotic vertebral collapse	44,386	N331.14
Collapse of cervical vertebra due to osteoporosis	45,736	N331H00
Osteoporosis + pathological fracture cervical vertebrae	48,772	N331A00
C3 vertebra closed fracture without spinal cord lesion	52,699	S100311
Closed fracture cervical vertebra, wedge	53,337	S100H00
Closed fracture of third cervical vertebra	60,593	S100300
Open fracture lumbar vertebra, wedge	65,302	S105100
C4 vertebra closed fracture without spinal cord lesion	67,358	S100411
[X]Collapsed vertebra in diseases classified elsewhere	73,900	Nyu6700
[X]Fracture of other specified cervical vertebra	94,638	Syu1500
Collapse of cervical vertebra	110,999	N331C11
Wrist/distal radius		
Wrist fracture–closed	203	S234.11
Closed reduction of fracture of wrist	5951	7K1LM00
Fracture at wrist and hand level	8056	S242.00
Wrist fracture–open	10,022	S235.11
Closed fracture‐subluxation of the wrist	17,921	S4C2.00
Fracture‐dislocation or subluxation of wrist	18,614	S4C.00
Closed fracture dislocation of wrist	34,429	S4C0.00
Open fracture dislocation wrist	42,844	S4C1.00
[X]Fracture of other and unspecified parts of wrist and hand	53,068	Syu6500
Open fracture‐subluxation of the wrist	59,219	S4C3.00
Closed fracture distal radius, extra‐articular, other type	19,058	S234D00
Closed fracture distal radius, intra‐articular, die‐punch	44,844	S234C00
Closed fracture distal radius, intra‐articular, other type	28,293	S234E00
Closed fracture of the distal radius, unspecified	1742	S234200
Open fracture distal radius, extra‐articular other type	53,698	S235D00
Open fracture distal radius, intra‐articular other type	63,588	S235E00
Open fracture distal radius, intra‐articular, die‐punch	104,070	S235C00
Humerus		
Fracture of humerus	517	S22.00
Fracture of upper end of humerus	2101	S226.00
Closed reduction of fracture of humerus	6379	7K1LF00
Open fracture of the proximal humerus	9420	S221.00
Fracture of humerus NOS	10,382	S22z.00
Closed fracture proximal humerus, greater tuberosity	11,044	S220300
Closed fracture of the proximal humerus	11,222	S220.00
Closed fracture proximal humerus, neck	11,313	S220100
Closed fracture of humerus NOS	19,186	S222000
Closed fracture of humerus, shaft	27,886	S222100
Closed fracture proximal humerus, head	28,739	S220400
Closed fracture proximal humerus, four part	29,137	S220700
Fracture of shaft of humerus	30,659	S227.00
Closed fracture of proximal humerus, anatomical neck	33,489	S220200
Closed fracture of humerus, shaft or unspecified part	36,464	S222.00
Closed fracture of proximal humerus not otherwise specified	38,353	S220z00
Closed fracture proximal humerus, three part	40,330	S220600
Closed fracture of proximal humerus, unspecified part	44,721	S220000
Open fracture of proximal humerus not otherwise specified	45,275	S221z00
Open fracture proximal humerus, greater tuberosity	48,239	S221300
Closed fracture of humerus, upper epiphysis	52,406	S220500
Open fracture proximal humerus, neck	53,622	S221100
Open fracture of proximal humerus, unspecified part	53,688	S221000
Closed fracture of humerus, shaft or unspecified part NOS	61,378	S222z00
Open fracture proximal humerus, head	70,486	S221400
Open fracture proximal humerus, three part	70,604	S221600
Open fracture proximal humerus, four part	70,653	S221700
Open fracture of proximal humerus, anatomical neck	71,207	S221200
Rib		
Closed fracture rib	280	S120.00
Closed fracture of rib, unspecified	7831	S120000
Fracture of rib	9688	S127.00
Multiple fractures of ribs	10,696	S127000
Closed fracture multiple ribs	11,004	S120900
Rib fracture NOS	11,378	S12z.11
Closed fracture of one rib	16,494	S120100
Multiple rib fractures	17,107	S29.12
Closed fracture of rib(s) NOS	28,244	S120z00
Cough fracture of ribs	28,538	S127100
Closed fracture of four ribs	34,197	S120400
Closed fracture of two ribs	40,533	S120200
Open fracture of two ribs	44,826	S121200
Open fracture rib	48,224	S121.00
Closed fracture of five ribs	55,424	S120500
Closed fracture of three ribs	56,384	S120300
Closed fracture of six ribs	65,484	S120600
Closed fracture of eight or more ribs	68,652	S120800
Open fracture multiple ribs	71,452	S121900
Open fracture of rib, unspecified	73,613	S121000
Open fracture of seven ribs	73,956	S121700
Closed fracture of seven ribs	90,494	S120700
Open fracture of rib(s) NOS	101,560	S121z00
Open fracture of one rib	112,701	S121100
Open fracture of four ribs	113,739	S121400
Pelvis		
Fracture or disruption of pelvis	738	S13.00
Closed fracture pelvis, multiple pubic rami‐stable	6667	S132100
Closed fracture pelvis, single pubic ramus	7004	S132000
Other or multiple closed fracture of pelvis NOS	11,639	S134z00
Fracture of lumbar spine and pelvis	12,406	S10B.00
Closed fracture pelvis, coccyx	14,834	S108.00
Closed fracture of pelvis NOS	28,375	S13y.00
Other or multiple closed fracture of pelvis	33,961	S134.00
Closed fracture pelvis, ischium	34,708	S134100
Closed fracture‐dislocation of pelvis	34,910	S4J0100
Closed fracture pelvis, multiple pubic rami‐unstable	46,592	S132200
Unspecified site		
Fragility fracture due to unspecified osteoporosis	11,503	N331M00
H/O: fragility fracture	18,731	14G6.00
[X]Unspecified osteoporosis with pathological fracture	18,825	NyuB800
Idiopathic osteoporosis with pathological fracture	27,597	N331600
Osteoporosis of disuse with pathological fracture	33,526	N331300
Postmenopausal osteoporosis with pathological fracture	38,395	N331B00
Postoophorectomy osteoporosis with pathological fracture	39,334	N331200
Drug‐induced osteoporosis with pathological fracture	46,894	N331500
[X]Other osteoporosis with pathological fracture	57,301	NyuB000
Postsurgical malabsorption osteoporosis with path fracture	68,019	N331400
Fragility fracture	93,497	N331N00
Minimal trauma fracture due to unspecified osteoporosis	93,705	N331M11

**TABLE A3:** Search Terms for prescriptions

Search term	Target
Bisphosphon	Bisphosphonate/Bisphosphonic acid
Disphosphon	Diphosphonate/Diphosphonic acid
Alendron	Alendronate/Alendronic acid
MK‐217	Alendronic acid (Alternative notation)
Ibandron	Ibandronate/Ibandronic acid
Risedron	Risedronate/Risedronic acid
Zolendron	Zoledronate/Zolendronic acid
Etidron	Etidronate/Etidronic acid
EHDP	Etidronate (alternative notation)
Strontium ranelate	Strontium ranelate
Sodium clondronate	Sodium clondronate
Tiludron	Tilundronate/Tilundronic acid
Denosumab	Denosumab
AMG 162	Denosumab (alternative notation)
Romosozumab	Romosozumab
AMG 785	Romosozumab (alternative notation)
Teriparatide	Teriparatide
Human parathyroid hormone	Human parathyroid hormone
Abaloparatide	Abaloparatide
BNF chapters	
060602	Bone protective medications
090604	Vitamin D supplementation
09050101	Calcium supplementation

**TABLE A4:** Table of bone protective medication prescriptions over 48 weeks following a fragility fracture

Timeline	Censored from study (N)	Number of participants^a^	Oral bisphosphonate	Strontium ranelate	Denosumab	Zoledronate	All
Before fracture 1	0	1823	217	6	2	0	225
(Percentage)			(11.9%)	(0.3%)	(0.1%)	(0.0%)	(12.3%)
Up to 16 weeks after	236	1587	572	17	5	3	597
(Percentage)			(36.0%)	(1.1%)	(0.3%)	(0.2%)	(37.6%)
Up to 32 weeks after	369	1454	653	18	9	3	683
(Percentage)			(44.9%)	(1.2%)	(0.6%)	(0.2%)	(47.0%)
Up to 48 weeks after	479	1344	678	18	12	3	711
(Percentage)			(50.4%)	(1.3%)	(0.9%)	(0.2%)	(52.9%)

*Note*: This table displays the cumulative frequency of each medication type and all bone protective medications, starting before the fracture event followed by 16 week intervals.

^a^ The number of people with available data within each time window for bone protective medication.

**TABLE A5:** Table of vitamin D and/or calcium supplement prescriptions over 48 weeks following a fragility fracture

Timeline	Censored from study (N)	Number of participants^a^	Supplementation^b^
Before fracture 1	0	1823	442
(Percentage)			(24.2%)
Up to 16 weeks after	181	1642	951
(Percentage)			(57.9%)
Up to 32 weeks after	269	1554	1054
(Percentage)			(67.8%)
Up to 48 weeks after	330	1493	1093
(Percentage)			(73.2%)

*Note*: This table displays the cumulative frequency of supplementation (vitamin D and/or calcium) starting before the fracture event followed by 16 week intervals.

^a^ The number of people with available data within each time window for supplementation (Vitamin D and/or calcium).

^b^ Supplementation refers to a prescription being received for vitamin D and/or calcium.

**TABLE A6:** Sensitivity analysis restricted to those with an initial diagnosis of Parkinson's disease

	Odds ratio (95% CI)	N	*p*‐value
Fracture type (unadjusted)			
Hip	ref	1521	
Vertebral	1.79 (1.31–2.43)		< 0.001
Wrist	0.64 (0.40–1.02)		0.06
Humerus	0.72 (0.50–1.04)		0.08
Rib	0.25 (0.13–0.46)		< 0.001
Pelvis	1.00 (0.57–1.73)		0.99
Unspecified	1.69 (1.24–2.30)		< 0.001
Multiple fractures	1.38 (0.61–3.12)		0.44
Fracture type adjusted for age			
Hip	ref	1521	
Vertebral	1.91 (1.40–2.61)		< 0.001
Wrist	0.72 (0.45–1.16)		0.18
Humerus	0.74 (0.51–1.08)		0.12
Rib	0.28 (0.15–0.53)		< 0.001
Pelvis	1.00 (0.57–1.75)		1.00
Unspecified	1.79 (1.31–2.44)		< 0.001
Multiple fractures	1.40 (0.61–3.19)		0.42
Fracture type adjusted for age and gender			
Hip	ref	1521	
Vertebral	2.04 (1.48–2.81)		< 0.001
Wrist	0.56 (0.34–0.91)		0.02
Humerus	0.64 (0.44–0.94)		0.02
Rib	0.31 (0.16–0.58)		< 0.001
Pelvis	0.85 (0.48–1.49)		0.56
Unspecified	1.53 (1.11–2.10)		0.01
Multiple Fractures	1.31 (0.56–3.02)		0.53
Cambridge multimorbidity score unadjusted			
0/1	ref	1521	
2	0.80 (0.48–1.36)		0.42
3	0.80 (0.48–1.33)		0.39
4	0.88 (0.53–1.45)		0.61
5	0.95 (0.58–1.57)		0.85
6	0.97 (0.58–1.63)		0.92
7+	0.87 (0.53–1.43)		0.58
Cambridge multimorbidity score adjusted for age			
0/1	ref	1521	
2	0.74 (0.44–1.27)		0.28
3	0.74 (0.44–1.23)		0.24
4	0.79 (0.48–1.31)		0.36
5	0.84 (0.50–1.40)		0.49
6	0.83 (0.49–1.41)		0.50
7+	0.76 (0.46–1.26)		0.29
Care home residence (Unadjusted)^a^			
Not living in care home	ref	1521	
Living in care home	0.75 (0.48–1.17)		0.20
Care home residence adjusted for age			
Not living in care home	ref	1521	
Living in care home	0.68 (0.44–1.07)		0.10
Care home residence adjusted for age and gender			
Not living in care home	ref	1521	
Living in care home	0.69 (0.44–1.08)		0.11
Care home residence adjusted for age, gender and CMS			
Not living in care home	ref	1521	
Living in care home	0.68 (0.43–1.08)		0.10

^a^ There are now 94 (6.2%) of patients living in a care home.

**TABLE A7:** Sensitivity analysis restricted to those with 48‐weeks of data before and after the index fracture

	Odds ratio (95% CI)	N	*p*‐value
Fracture type (Unadjusted)			
Hip	ref	765	
Vertebral	1.64 (1.05–2.56)		0.03
Wrist	0.58 (0.32–1.05)		0.07
Humerus	0.67 (0.39–1.13)		0.13
Rib	0.22 (0.10–0.48)		<0.001
Pelvis	0.56 (0.25–1.27)		0.17
Unspecified	1.57 (1.05–2.36)		0.03
Multiple Fractures	1.31 (0.37–4.63)		0.67
Fracture type adjusted for age			
Hip	ref	765	
Vertebral	1.73 (1.10–2.71)		0.02
Wrist	0.62 (0.34–1.14)		0.13
Humerus	0.69 (0.40–1.17)		0.17
Rib	0.24 (0.11–0.52)		<0.001
Pelvis	0.57 (0.25–1.30)		0.18
Unspecified	1.61 (1.07–2.42)		0.02
Multiple Fractures	1.36 (0.38–4.84)		0.64
Fracture type adjusted for age and gender			
Hip	ref	765	
Vertebral	1.78 (1.12–2.82)		0.01
Wrist	0.50 (0.27–0.94)		0.03
Humerus	0.60 (0.35–1.03)		0.06
Rib	0.26 (0.12–0.57)		< 0.001
Pelvis	0.48 (0.21–1.10)		0.08
Unspecified	1.44 (0.95–2.19)		0.09
Multiple Fractures	1.30 (0.36–4.69)		0.69
Cambridge multimorbidity score unadjusted			
0/1	ref	765	
2	0.66 (0.29–1.48)		0.31
3	0.85 (0.40–1.81)		0.67
4	1.02 (0.48–2.16)		0.96
5	0.85 (0.39–1.84)		0.68
6	1.16 (0.53–2.56)		0.71
7+	1.08 (0.51–2.29)		0.84
Cambridge multimorbidity score adjusted for age			
0/1	ref	765	
2	0.60 (0.26–1.37)		0.23
3	0.79 (0.36–1.70)		0.54
4	0.90 (0.42–1.93)		0.78
5	0.76 (0.34–1.66)		0.48
6	0.97 (0.43–2.17)		0.93
7+	0.91 (0.42–1.95)		0.80
Care home residence (unadjusted)^a^			
Not living in care home	ref	765	
Living in care home	0.54 (0.24–1.18)		0.12
Care home residence adjusted for age			
Not living in care home	ref	765	
Living in care home	0.48 (0.22–1.07)		0.07
Care home residence adjusted for age and gender			
Not living in care home	ref	765	
Living in care home	0.47 (0.21–1.05)		0.07
Care home residence adjusted for age, gender and CMS			
Not living in care home	ref	765	
Living in care home	0.44 (0.20–1.00)		0.05

^a^ There are now 31 (4.1%) of patients living in a care home.
